# *Enterocytozoon hepatopenaei* Infection in Shrimp: Diagnosis, Interventions, and Food Safety Guidelines

**DOI:** 10.3390/microorganisms12010021

**Published:** 2023-12-22

**Authors:** Thenmoli Govindasamy, Subha Bhassu, Chandramathi Samudi Raju

**Affiliations:** 1 Animal Genetics and Genome Evolutionary Laboratory (AGAGEL), Department of Genetics and Microbiology, Institute of Biological Sciences, Faculty of Science, University of Malaya, Kuala Lumpur 50603, Malaysia; 2 Terra Aqua Laboratory, Centre for Research in Biotechnology for Agriculture (CEBAR), Research Management and Innovation Complex, University of Malaya, Kuala Lumpur 50603, Malaysia; 3 Department of Medical Microbiology, Faculty of Medicine, University of Malaya, Kuala Lumpur 50603, Malaysia; chandramathi@um.edu.my

**Keywords:** shrimp diseases, microsporidia, lifecycle, biosecurity

## Abstract

The emergence of disease in shrimp has governed much concern in food safety and security among consumers with the recent reports on hepatopancreatic microsporidiosis (HPM) caused by *Enterocytozoon hepatopenaei* (EHP). The microsporidians present in shrimp remain a silent pathogen that prevents optimal shrimp growth. However, the biggest threat is in its food safety concerns, which is the primary focus in ensuring food biosecurity and biosafety. Hence, the objective of this review is to summarise the current knowledge of EHP and its infection in shrimp with food safety concerns. This paper provides an analysis of the diagnostic methods for detecting EHP infections in shrimp aquaculture. Interventions with current molecular biology and biotechnology would be the second approach to addressing EHP diseases. Finally, a systematic guideline for shrimp food safety using diagnostic and intervention is proposed. Thus, this review was aimed to shed light on effective methods for the diagnosis and prevention of EHP infection in shrimp. We also include information on molecular and genomics tools as well as innate immune biomolecules as future targets in the intervention strategies on the microsporidsosis life cycle in shrimp and its environment. Overall, this will result in reduced disease outbreaks in shrimp aquaculture, ensuring the shrimp food safety in the future.

## 1. Introduction

Shrimp aquaculture, also known as shrimp farming, is a business that cultivates various types of marine shrimps and prawns for human consumption, with farmed shrimp now representing more than half of the world’s total shrimp supply [1]. According to the World Wildlife Fund (WWF), 80% of shrimp farming is dominated by two penaeid shrimp species, *Penaeus monodon* (giant tiger prawn) and *Penaeus vannamei* (white leg shrimp) [2]. Prior to the year 2000, the most widely grown shrimp species in Asia was *Penaeus monodon*, and this began to change at that time, as domesticated and specific pathogen-free (SPF) *Penaeus vannamei* became the preferred species for shrimp farmers worldwide [3]. However, increased disease outbreak in shrimp has raised questions on the sustainability of shrimp farming, which has grown to be a barrier to the production of farmed shrimp on a worldwide scale [4]. A newly emerging disease, hepatopancreatic microsporidiosis (HPM) caused by *Enterocytozoon hepatopenaei* (EHP), has raised concerns in the shrimp aquaculture industry. This disease is named HPM mainly because the target organ of EHP is the hepatopancreas, in which the microsporidians infect the hepatopancreatic tubule epithelial cells of crustaceans by altering biochemical parameters such as albumin, aspartate transaminase (AST), aspartate transaminase (ALT), and alkaline phosphatase [4]. HPM causes delayed growth and wide size differences in cultivated shrimp, making the causal agent, EHP, an economically concerning pathogen in shrimp aquacultures [5].

EHP is a specialised, unicellular, spore-forming parasite of humans, animals, insects, and crustaceans that is a member of the family *Enterocytozoonidae* and phylum Microsporidia [6,7]. EHP infection retards the growth of shrimp but does not cause mortality or gross signs of disease [8]. However, EHP infection prevents the optimal growth of shrimp, resulting in an economic loss for the country. Moreover, EHP proliferates within the cytoplasm of infected cells, and the histopathology of EHP-infected shrimp reveals irregular or regular basophilic inclusion bodies within the cytoplasm, regardless of the presence of spores [9]. To date, no treatment is available to cure this problem caused by EHP in shrimp, as EHP produces highly resistant and stable spores [4]. Hence, this problem might leave an impact on food safety, as shrimp is a seafood that can cause human illnesses upon consumption. 

Thus, this review provides a summary of the diagnosis of HPM caused by EHP in relation to food safety concerns with further discussion on addressing EHP diseases using current molecular biology, and it proposes a systematic guideline for shrimp food safety using diagnostic and intervention tools.

## 2. EHP Diagnostic Methods 

### 2.1. Histopathology

#### 2.1.1. Microscopy 

**Figure 1:**
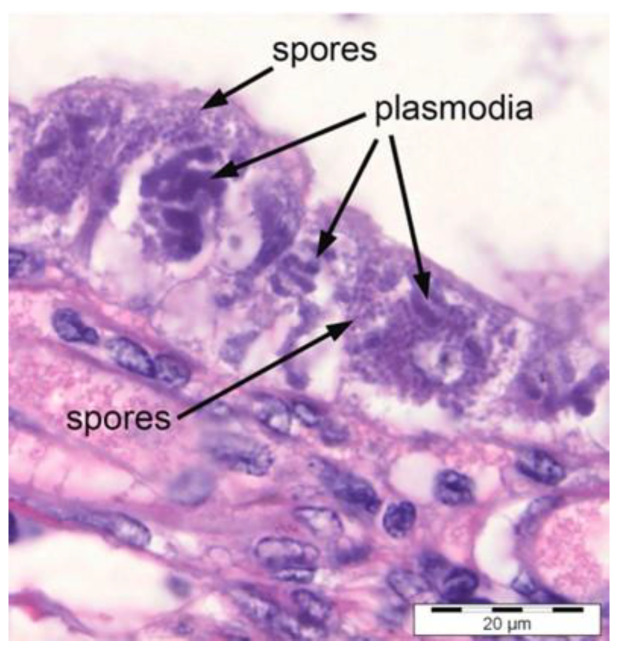
H&E-stained HP tissue showing EHP spores and plasmodia [6].

EHP can be diagnosed by using a microscopic method using haematoxylin–eosin (H&E)-stained tissue sections. This method can be carried out by carefully observing spores in hepatopancreatic tissue and faecal samples using microscopic analysis [4]. For light microscopy analysis, the shrimp hepatopancreases need to be fixed in a fixative such as Davidson fixative, processed for histology, and stained with H&E [10]. Histopathological analysis of EHP infections reveals EHP life stages in hepatopancreatic (HP) tubule epithelial cells and free spores that have been released into the HP tubule lumens from lysed epithelial cells [6]. This was proven by a study [10] in which the histological analysis showed EHP life stages, mature spores, and severe necrotic epithelium and HP tubules as shown in Figure 1.

However, the sensitivity of the H&E approach falls short of the criteria in the EHP preventive plan because spores cannot be distinguished easily from normal host cells in low-level infections [6,11]. Thus, the development of in situ hybridisation (ISH) is needed to confirm the results obtained. 

#### 2.1.2. In Situ Hybridisation (ISH)

**Figure 2:**
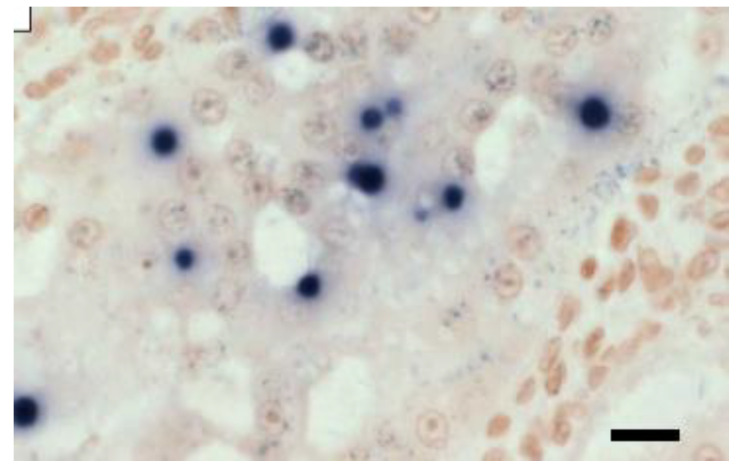
In situ hybridisation of HP tissue of *Penaeus vannamei* with a digoxigenin-labelled EHP probe [13]. Scale bar = 25 µm.

The DIG-labelled 18SrRNA gene probe is employed in ISH assays to identify EHP, as it allows for the assessment infection severity because all EHP life stages can be seen, even at low magnification levels [11,12]. In addition, ISH was reported to be more sensitive and precise in detecting causative agents, as cells with no visible evidence of microsporidian spores from histological analysis can be determined to be positive [12] as shown in Figure 2.

However, complex ISH techniques are unsuitable for practical use in farm settings because they are time-consuming and not practical enough to be used as a regular approach to detect EHP.

### 2.2. Molecular Detection Methods 

#### 2.2.1. Polymerase Chain Reaction (PCR)

PCR remains a common technique used in the diagnosis of shrimp diseases, as it is simpler and more cost-effective. The types of PCR methods used in EHP detection are one-step PCR [14], qPCR [15], and nested PCR [5]. In this case, one-step PCR is easier to execute and needs only a set of primers; however, the detection threshold frequently varies between 1000 and 10,000 copies per reaction, which is insufficient to detect infection spread via carriers [6]. In a study conducted on developing a PCR assay for the effective detection of EHP and investigation of EHP prevalence in Shandong Province, China, a pair of primers amplifying 358 base pairs of an EHP DNA fragment was designed, and it was shown to have the ability to detect EHP at a copy number as low as 2 × 10^1^ [14]. This method was reported to be more sensitive and specific compared to previous EHP PCR assays. Moreover, the newly developed PCR assay can be used to identify EHP in numerous shrimp samples in a timely and effective manner.

Meanwhile, nested PCR employs two sets of primers to successively amplify the target, which yields at least 10 times the sensitivity of its one-step equivalent [6]. As a result, this leads to the ability to detect low-level infections. Because existing PCR methods targeting EHP SSU rRNA were found to give false positive test results due to the cross-reactivity of the SSU-PCR primers with DNA from closely related microsporidia, the nested PCR method was developed for the detection of the spore wall protein (SWP) gene of EHP [5]. This method of nested PCR was found to successfully distinguish EHP and did not yield false positive results from related microsporidia. Thus, it is recommended to design PCR applications around an SWP gene or genes with high sequence diversity in aquatic microsporidia. Moreover, a modified method of nested PCR to detect EHP in *Macrobrachium rosenbergi*, giant freshwater prawn was carried out in which the primers were redesigned to detect the distinct strain of EHP. This is because the former primers could not amplify the EHP SWP1 gene; thus, the nested SWP-PCR method was altered, and the new primers showed high specificity and sensitivity [16]. Overall, this study’s method was proposed to be beneficial for investigating EHP mutants in epidemiological studies, and SWP1 gene mutation allows for a better understanding of the molecular mechanisms by which EHP adapts to diverse hosts.

In addition, qPCR, which is a quantitative method, is required to assess the severity of infection and the progression of disease because qPCR is used to quantify pathogens based on the linear relationship between the logarithm of template copy number and the number of PCR cycles needed to attain a threshold [6,17]. In a study conducted to detect and quantify EHP in infected shrimp *Litopenaeus vannamei*, a SYBR Green I fluorescent qPCR assay was designed based on the polar tube protein 2 (PTP2) gene [15]. The study found that the efficiency of amplification was 102%, and the qPCR technique was determined to be highly sensitive, specific, and repeatable. However, the fundamental disadvantage of qPCR and its variants is the need for costly qPCR equipment, considerable liquid handling, and employees with advanced molecular biology expertise [6]. 

#### 2.2.2. Recombinase Polymerase Amplification (RPA)

RPA is a rapid and simple isothermal amplification technique. This technique can amplify DNA in shorter reaction time and requires only one pair of primers [6]. In addition, RPA is also a reliable and effective on-site detection technology [6,18]. A real-time RPA assay was established by combining fluorescence analysis with the RPA system for the rapid detection of EHP infection in shrimp [18]. According to the findings of the study, the detection was done in 10 min with good specificity using this technique, and the detection results for actual clinical samples were 100 percent in agreement with the established nested PCR technique [18]. Overall, the RPA assay can be widely used in detecting EHP infection in remote areas, as it is simple and reliable. 

#### 2.2.3. Loop-Mediated Isothermal Amplification (LAMP)

The LAMP assay is quick to detect EHP with a constant reaction temperature and by using a simple dry bath without requiring any technical expertise or costly equipment such as a thermocycler [6,19]. Recently, an improved colorimetric EHP LAMP diagnostic assay was developed with primers specific to EHP spore wall protein (SWP) gene for the visual detection of EHP [19]. In this study, hydroxy naphthol blue (HNB) or phenol red dye was used to achieve the visual detection of LAMP amplicons without opening the tubes to prevent contamination. Moreover, the EHP LAMP assay showed 95.31% sensitivity, 98.98% specificity, and a kappa value of 0.948 compared to the gold standard, SWP-PCR [20]. Hence, the LAMP assay is user-friendly and has great potential to be performed at farm sites. The Table 1 shows all primers used in molecular detection techniques in EHP.

## 3. Suggested Future EHP Detection Methods

Apart from the available methods, many of the other latest diagnostic techniques can be utilised for the effective detection of EHP as meeting the expanding diagnosis and control demand becomes increasingly difficult. For example, digital PCR (dPCR), which is the third generation of the PCR, is gaining popularity due to its capacity to completely quantify pathogens while maintaining excellent selectivity, simplicity, accuracy, and speed. In addition, the dPCR contributes to measuring low nucleic acid levels and inhibitor resistance, and it enables pathogenic detection with zero tolerance [23]. Currently, the dPCR is also used to detect fungi, indicating that it is a valuable tool for detecting viruses, bacteria, parasites, fungi, and chlamydia. 

Recently, a sensitive and precise duplex droplet digital PCR (ddPCR) method was carried out to simultaneously detect and quantify EHP and *Vibrio parahaemolyticus* acute hepatopancreatic necrosis disease (VPAHPND). The ddPCR showed 10-fold higher sensitivity compared to the qPCR, in which the sensitivity levels for EHP and VPAHPND were 2.3 copies/μL and 4.6 copies/μL, respectively [24]. Hence, the ddPCR is recommended for use in detecting EHP because it is expected to benefit the investigation of complex genomic targets and usher in a new era of pathogenic diagnostics.

Next-generation sequencing (NGS) is a high-throughput sequencing technique that provides new ways of detecting microorganisms beyond microbial culture-based methods [25]. Moreover, NGS can be used to monitor microbiota changes within the animal host and in the environment in order to utilise them as markers or predictors of animal health status. In addition, fungal identification using NGS is more accurate and accessible because this technology is ideal for hostile culture and microbial infections such as fungi. Therefore, NGS can be suggested to be used in EHP detection, as it could provide a method to observe changes in the microbiota of EHP throughout the duration of infection and to enhance our knowledge on EHP pathogenesis beyond direct host–EHP interactions [6].

Environmental DNA (eDNA) is DNA found in environmental samples such as soil, water, sediments, or air that organisms have released into their environment via secretions and discharges such as mucus, urine, blood, gametes, saliva, shed skin cells, faeces, hair, body remains, etc. In addition, the term “environmental DNA” refers to the ability to extract microbial nucleic acids directly from environmental samples [26]. eDNA technology recently became widely used as a new aquatic organism survey tool for species detection, biodiversity evaluation, and population abundance determination [27]. Other than that, eDNA technology offers benefits such as convenient sampling, cost-efficiency, and high sensitivity compared to traditional methods. 

Additionally, biosensors can also be used as an EHP detection method because they integrate a biological recognition element with a physicochemical reporter to detect a variety of analytes such as proteins, metabolites, DNA, RNA, etc. [6]. For example, an immunosensor designed based on a quartz crystal microbalance (QCM) to detect *Vibrio harveyi*, which causes illness and mortality in commercial shrimp farms, could be utilised to detect *Vibrio harveyi* in a range of 10^3^–10^7^ CFU/mL [28]. However, due to the expensive materials required, the complexity of data interpretation, and the sensitivity to biological matrices, this method is not desirable. 

## 4. Interventions in Managing EHP 

The most important aspects in managing EHP would be biosecurity measures and good management practices in farms. First, clean brood stock that supplies eggs and nauplii for hatcheries must be carefully procured to prevent disease carriers from entering the culture system. It is highly advised to adhere to strict biosecurity and good pond management protocols in shrimp aquaculture, such as chlorination, de-chlorination of water, liming, drying, and ploughing, to prevent EHP infection because it is difficult to eradicate the disease once spores are present in ponds [4]. However, the inhibition of spore extrusion can be an effective method to control EHP infections. EHP spore extrusion can be inhibited by inactivating the spores upon heating at 75 °C or freezing at −20 °C. Moreover, quicklime (CaO) can be used to treat the pond before stocking to spore extrusion. This is because low pH levels were proven to inactivate spores. In a study conducted in which spores were incubated in buffer at pH levels of 4, 7, and 9, they showed germination rates of 5%, 10%, and 90%, respectively [6]. 

Furthermore, shrimp from aquacultures should be screened for EHP using molecular techniques on a regular basis [6]. It is necessary to sample different parts of the pond, as infection may be uneven. eDNA protocols can be used by sampling the water and soil from shrimp aquacultures to confirm the presence of an EHP infection. The eDNA technique reduces the time associated with sample collection and processing, as it does not require handling animals. 

In addition, boosting the immune system of shrimp can be considered as a method to control disease outbreaks in aquacultures. Feed additives containing zinc and selenium can promote the immune health of shrimp in which zinc particularly speeds up the wound-healing process. Moreover, shrimp, as invertebrates, depend mostly on their innate immune systems to battle invading diseases such as hepatopancreatic microsporidiosis (HPM), acute hepatopancreatic necrosis disease (AHPND), etc. Therefore, developing transgenerational innate immunological memory in shrimp may allow the production of offspring with greater resistance to infectious illnesses. Earlier exposure to live or killed bacteria or viral proteins was shown to improve protection during a secondary infection and increase survival rate in shrimp [29]. Recently, a study was conducted by inducing transgenerational innate immune memory against *Vibrio* infections in a brine shrimp (*Artemia franciscana*) model in which one parental generation was exposed to live or dead *Vibrio parahaemolyticus* PV1, and another parental generation was exposed to live or dead *Vibrio campbellii* LMG2136. It was reported that offspring of primed F_0_ parents exhibited innate immunological memory compared to those of non-primed control F_0_ parents because they had considerable defences against future *Vibrio* infections [29]. As a result, raising trained immunity in shrimp could provide crucial support to control disease and to develop a sustainable aquaculture.

Moreover, vaccines and immunostimulants are preventative measures that are used to strengthen the host’s immune system. In this case, shrimp must rely on their innate immune system because they lack an adaptive immune system. Hence, creating vaccinations for them is time-consuming, expensive, and tedious. Meanwhile, immunostimulants are chemicals that effectively stimulate the host’s non-specific defensive system in order to battle invading microorganisms. Thus, immunostimulants could be a promising approach for shrimp health management, as they compensate for the discrepancies in vaccine usage and give a reasonable solution in terms of shrimp immunity [30]. Immunostimulants are preferred over injecting antibiotics into shrimp because the biomagnification and bioaccumulation of antibiotic residues in food chains and food webs can cause allergies, toxicity, and resistance in humans. The use of immunostimulants has no negative effects on the environment because no toxic residues are accumulated, and it is also simple to administer immunostimulants to larvae and shrimp. In general, shrimp disease prevention and control both require an integrated strategy in which our understanding of shrimp immunity must be enhanced. 

Moreover, infected shrimp can transmit EHP to humans and cause diseases. To better understand the life cycle, pathogenicity, host immune responses, and pathobiology of fungal infections, gene editing tools such as CRISPR-Cas and RNA interference (RNAi) can be utilised. Genome editing has improved our ability to understand how genetics affect disease by promoting the development of more precise cellular and animal models of pathological processes [31]. 

The development of the CRISPR/Cas tool is a useful addition to gene editing technology for pathogenic fungi, especially in diploid fungal species that lack meiosis and transfecting plasmids [32]. This is due to the fact that CRISPR/Cas has evolved into a ground-breaking biotechnology and molecular biology tool that allows us to precisely perform nucleic acid detection, gene expression regulation, genomic, epigenomic, and RNA editing processes in a range of organisms. The usage of various types of vectors and sgRNA/Cas9 complexes to deliver the components of the CRISPR system into fungal cells makes it possible to modify gene expression in many fungal species [33]. 

In the realm of biotechnology, prime editing is the most recent gene-editing technique developed, which locates and modifies the points in the gene that need to be altered without damaging the double-stranded DNA at the target point [33]. Other than that, many studies are being conducted to develop new antifungal drugs because pathogenic fungi can cause infections in immunocompromised humans due to HIV, organ transplantation, cancer chemotherapy, etc. [34,35]. Overall, knowledge of these interventions can be applied in preventing EHP infection in shrimp aquaculture to ensure food safety. 

## 5. Food Safety

The aforementioned EHP detection techniques are critical for distinguishing between healthy and diseased shrimp in order to assure food safety in downstream consumption. Immunocompromised and immunodeficient people, such as AIDS patients, are susceptible to infection by EHP, as it is closely related to *Enterocytozoon bieneusi*, a species known to infect AIDS patients. Despite the lack of evidence that EHP infects other animals than shrimp, detecting EHP in shrimp is critical for human health [15]. This is due to the fact that infected shrimp exhibit no clear clinical symptoms over a short period of time, and healthy shrimp may become infected with EHP by cohabiting with diseased shrimp. In addition, biochemical assays and 16S rRNA analyses are proposed as appropriate diagnostic methods to determine shrimp gut microbiome changes and health status in order to ensure food safety [36]. As a result, it is critical to develop an effective approach to detect EHP infection in shrimp, particularly in the early stages of infection. 

Recently conducted studies suggest that exotic viral infections can be transmitted to cultivated shrimp stocks through frozen prawn products processed and packaged for human consumption. However, it was reported that there is no epidemiological evidence in the scientific literature or in public databases indicating that shrimp disease outbreaks in farms or in the wild fishery were caused by processed and packed shrimp for human consumption [37]. Although there is no evidence of shrimp infection being transmitted to human consumption, it is suggested that preventive steps should be implemented to assure food safety. Therefore, it is vital to investigate transmission pathways of EHP in the shrimp–human food chain in order to assess food chain safety [15]. 

### Systematic Guidelines for Shrimp Food Safety

It is crucial to follow systematic guidelines for shrimp food safety to ensure that the aquaculture sector produces safe food for consumption. There are five important aspects of the food chain approach to food safety [38]. First, food safety should be incorporated with risk analysis, which comprises risk assessment, risk management, and risk communication. Risk analysis involves identifying causative factors capable of causing adverse health impacts that may be present in aquaculture products. The causative agents include biological agents such as bacteria, virus, fungi, and chemical agents such as pesticides. For example, bacteria such as *Vibrio parahaemolyticus*, *Vibrio cholerae*, *Vibrio vulnificus*, *Salmonella* sp., and viruses such as norovirus and hepatitis A virus can be found in molluscan shellfish, shellfish, fish, and other aquaculture products. [38].

Moving on to the next process, risk assessment entails recording the sources of contamination, their frequency, and their concentration as well as an evaluation of the likelihood and likely concentrations at which they will be ingested. Information on the pathogen and the food, including the presence of microbes, pH level, nutritional content, and, ultimately, consumer food consumption habits, are used to carry out this process. Next, identifying available management alternatives, choosing the optimal option, taking relevant safety standards into consideration, and making the final management decision are all parts of risk management. In this case, it is necessary to maximise the efficacy, efficiency, technological viability, and practicality of food control measures at various stages of the food chain. Furthermore, all facets of communication between risk assessors, risk managers, and the public are included in the practical application of risk communication in connection to food safety. The ultimate goal of risk communication is to share the knowledge, attitudes, beliefs, practises, and views of interested parties regarding the dangers of eating certain foods and other relevant issues. 

Next, the aspect of traceability from the primary producer up to food processing and distribution to the consumer should be enhanced. Furthermore, to obtain comparable levels of protection against foodborne risks, food safety standards must be standardised, and food safety systems must be equivalent. Lastly, empowering food safety management requires a greater emphasis on risk prevention at the source along the whole food chain from farm to plate. Risk prevention in shrimp aquaculture can be done mainly by stocking post-larval shrimp from a specific pathogen-free brood stock, which minimises the effects of illness as a biosecurity and disease control measure [39].

A study conducted by Centre for Food Safety (CFS) in Hong Kong revealed that food poisoning occurrences are caused by causative organisms such as *Vibrio parahaemolyticus* and *Salmonella* sp., which were identified in raw shrimp sashimi and other foods. Hence, it is proven that raw foods are at higher risk of bearing such organisms, and that cooking seafood thoroughly is the greatest way to minimise the risk of foodborne illness by eliminating the pathogenic microorganisms present in the seafood. People with immunocompromised systems, pregnant women, nursing mothers, and elderly people should avoid consuming raw or inadequately prepared shellfish, as they are more susceptible to foodborne infections due to their weakened immune systems [40]. This also applies to foods at risk of EHP infection, as consuming food containing raw shrimp, such as sushi, that has EHP infection can negatively impact human health because EHP can be transmitted to the human body and cause diseases. Parasite infections in humans can cause various forms of gastrointestinal distress such as chronic diarrhoea, bloating, gas, nausea, and poor nutritional absorption leading to weight loss [41]. Although gastrointestinal distress is the most typical symptom of a parasite infection, they also can infect any organ system and result in keratitis, myositis, sinusitis, and encephalitis [41]. Hence, investigations of EHP and its impact on food safety are fundamental, as this issue is rarely addressed.

## 6. Conclusions

In conclusion, EHP has evolved as one of Asia’s most significant infections, causing HPM in cultivated white leg shrimp *Penaeus vannamei* [6]. Shrimp diseases have a significant effect on shrimp cultivation, and production sustainability is reliant on the balance between the environment, disease prevention through pathogen diagnostics and epidemiological surveys, and shrimp health [4]. Because there is no treatment available for EHP to-date, prevention is the greatest line of defence against EHP, especially considering the lack of a cost-effective, farm-scale therapy. Overall, this study will aid in the understanding of EHP and its diagnosis as well as of therapeutic protocols for EHP control, which will contribute to enhanced environmental quality and food safety. As a result, proper management and guidelines must be followed in shrimp farming systems, and extensive research on the diagnosis of EHP infection is required to assure food safety and prevent the transmission of EHP to humans.

## Figures and Tables

**Table 1:** List of primer sequences used in molecular detection of EHP in shrimp.

Method	Primer Name	Primer Sequence (5′–3′)	Reference
**One-step PCR** - SSU rRNA	EHP-510F	GCCTGAGAGATG GCTCCCACGT	[12]
	EHP-510R	GCGTACTATCCCCAGAGCCCGA	
**qPCR** - PTP2	EHP-PTP2-F	GCAGCACTCAAGGAATGGC	[15]
	EHP-PTP2-R	TTTCGTTAGGCTTACCCTGTGA	
**Nested PCR** - EhSWP	SWP_1F	TTGCAGAGTGTTGTTAAGGGTTT	[5]
	SWP_1R	CACGATGTGTCTTTGCAATTTTC	
	SWP_2F	TTGGCGGCACAATTCTCAAACA	
	SWP_2R	GCTGTTTGTCTCCAACTGTATTTGA	
	SWP_2F’	GCAGAGTGTTGTTAAGGGTTTAAG	[16]
	SWP_2R’	GCTGTTTGTCWCCAACTGTATT	
**Nested PCR** - SSU rRNA	ENF779	CAGCAGGCGCGAAAATTGTCCA	[21]
	ENR779	AAGAGATATTGTATTGCGCTTGCTG	
	ENF176	CAACGCGGGAAAACTTACCA	
	ENR176	ACCTGTTATTGCCTTCTCCCTCC	
**RPA** - SSU rRNA	F2	CATTGAGTTTGTTGAGAGTAGCGGAACGGAT	[22]
	R2	CTAAGAGCATCGCTTTCGCCTCCGTTGGTC	

## Data Availability

Not applicable.
